# Synthesis and Characterization of Nanoporous ZnO Films by Controlling the Zn Sublimation by Using ZnO/Zn Precursor Films

**DOI:** 10.3390/ma15165509

**Published:** 2022-08-11

**Authors:** Yazmin Mariela Hernández-Rodríguez, Primavera Lopez-Salazar, Gabriel Juarez-Diaz, Gabriel Romero Paredes-Rubio, Ramón Peña-Sierra

**Affiliations:** 1 Programa de Doctorado en Nanociencias y Nanotecnología, CINVESTAV-Instituto Politécnico Nacional, Av. IPN 2508, Mexico City 07360, Mexico; 2 Centro de Investigación en Dispositivos Semiconductores, Instituto de Ciencias, Benemérita Universidad Autónoma de Puebla, Ciudad Universitaria, Puebla 72570, Mexico; 3 Facultad de Ciencias de la Computación, BUAP, Puebla 72570, Mexico; 4 Departamento de Ingeniería Eléctrica, Sección de Electrónica del Estado Sólido (SEES) CINVESTAV-IPN, Av. IPN 2508, Mexico City 07360, Mexico

**Keywords:** zinc oxide, nanoporous ZnO films, Zn sublimation, hexagonal nut-like structures

## Abstract

A reliable process for the formation of nanoporous ZnO films supported on amorphous quartz and (100) silicon substrates via the processing of ZnO/Zn precursor films is reported. The process is based on the sublimation mechanism of Zn implemented in a novel ZnO/Zn precursor film to produce a nanoporous film. A scanning electron microscopy analysis of the nanoporous ZnO films’ surfaces revealed the presence of ZnO nano-features with round tips; in contrast, the nanoporous ZnO films supported on (100) Si substrates showed hexagonal nut-like nanostructures. The crystallite size of the nanoporous ZnO films decreased as the sublimation temperature was increased. X-ray photoelectron spectroscopy studies demonstrated that formations of oxygen vacancies were produced during the processing stages (as the main structural lattice defects in the ZnO nanoporous films). The analysis of the photoluminescence response confirmed that the active deep-level centers were also related to the oxygen vacancies generated during the thermal processing of the ZnO/Zn precursor films. Finally, a qualitative mechanism is proposed to explain the formation of nanoporous ZnO films on quartz and crystalline Si substrates. The results suggest that the substrates used have a strong influence on the nanoporous ZnO structures obtained with the Zn-sublimation-controlled process.

## 1. Introduction

Zinc oxide (ZnO) is a well-known metal oxide semiconductor belonging to the II–VI group with a hexagonal wurtzite structure. It exhibits outstanding physicochemical properties and relevant technological applications. The advantageous properties of ZnO can be illustrated by the wide bandgap energy of 3.37 eV at room temperature with an excitonic binding energy of 60 meV [1]. This allows diverse applications, such as gas sensing [2], photocatalysis [3], photovoltaic devices [4,5,6], hydrogen storage [7], and light-emitting diodes [8]. In addition, ZnO has high biocompatibility [9,10].

Controlling Zn vapor pressure during film growth also offers the potential to produce functional materials, e.g., nanoporous ZnO films with high surface-area-to-volume ratios for gas-sensing applications. For this application, nanoporous ZnO films can be fabricated by appropriately controlling the Zn vapor pressure. In addition, Zn films can be transformed into highly extended ZnO surfaces with high concentrations of structural defects, such as O vacancies (V_O_), Zn vacancies (V_Zn_), and interstitial Zn (Zn_i_) [11,12,13,14,15,16,17,18,19,20,21,22]. For accomplishing this, different methods have been proposed based on techniques such as pulsed-laser deposition (PLD), magnetron sputtering, electrodeposition, metal–organic chemical vapor deposition (MOCVD), and thermal oxidation of zinc [23,24,25,26].

The potential of Zn films to produce highly extended ZnO surfaces is based on the possibility of controlling the Zn pressure in the ZnO/Zn interface, which has been used for an extended period [27,28,29,30]. Such a method was studied by Anthrop and Searcy [31]; they were able to stabilize the Zn vapor pressure by adding water vapor and carbon dioxide to the working atmosphere. Recently, several studies have reported the formation of distinct tridimensional ZnO nanostructures with controlled geometries by regulating the Zn vapor pressure [32,33]. Even though several efforts have been made by researchers to achieve nanoporous ZnO thin films, innovative strategies are still required to produce highly dense nanoporous ZnO films in extensive areas.

Herein, we report an accessible and reproducible method for synthesizing nanoporous ZnO films with controlled morphologies. The method is based on the controlled sublimation of Zn in a novel ZnO/Zn precursor film at different temperatures under an atmosphere with a low O_2_ concentration. The advantage of using the precursor film is the possibility to work in a wide range of processing temperatures. Based on the results obtained in this study, a qualitative mechanism is proposed to explain the formation of nanoporous ZnO films on quartz and crystalline Si substrates.

## 2. Materials and Methods

### 2.1. Materials and Chemical Reagents

In this study, metallic Zn (purity: 5 N; Kurt J. Lesker) was used to deposit Zn nanofilms on Si and quartz substrates via the DC-sputtering deposition technique. Quartz (Quartz Slides, L4470, Agar Scientific, Stansted, UK) and (100) oriented p-type Si wafers (with resistances in the range of 2–5 Ω-cm) were used as the substrates; each was 1.5 × 1.5 cm^2^ in area. The quartz substrates were subsequently cleaned using xylene, acetone, and propanol in an ultrasonic bath, rinsed with deionized water, and, finally, dried under a filtered N_2_ atmosphere. The Si substrates were cleaned using the standard procedure of the Radio Company of America (RCA) [34]. For all of the experimental processes, chromatographic-grade gases were used, in addition to nitrogen (N_2_) and synthetic air consisting of a mixture of nitrogen and oxygen (N_2_/O_2_, 70%/30%). The sputtering deposition was performed by using an Intercovamex instrument (model X1). The working pressure was 5.5 mTorr, and it was controlled with ultra-high-purity (UHP) Ar gas; a continuous value of 30 W was set for 20 min to grow the zinc (Zn) films.

### 2.2. Synthesis of ZnO/Zn Precursor Films

The ZnO/Zn precursor films were produced through thermal oxidation of Zn at low temperatures under atmospheric pressure by following the procedure described in [35]. A brief description of this previously reported procedure is as follows: The nanometric ZnO films were produced by oxidizing the deposited Zn films at 350 °C for 30 min in a spare furnace equipped with a fused quartz chamber under a constant flow of 20 cm^3^/min of chromatographic N_2_. According to the supplier data, the N_2_ gas contained less than 5 ppm of O_2_ and H_2_O as impurities, which resulted in the growth of a ZnO film with a thickness of less than 5 nm, leading to the formation of a ZnO/Zn precursor film.

### 2.3. Synthesis of Nanoporous ZnO Films

Nanoporous ZnO films were produced by controlling the Zn sublimation of the ZnO/Zn precursor film at different temperatures. In this study, the processing temperature was fixed at three different values (600, 700, and 800 °C) to enhance the Zn sublimation rate. The synthesis of nanoporous ZnO films was performed under dry air (N_2_/O_2_, 70%/30%) for a time of 1 h. After finishing the thermal process, the samples were removed from the furnace and cooled at room temperature in laboratory conditions. The resulting ZnO films supported on quartz substrates were labeled as Q_1_, Q_2_, and Q_3_, and those supported on Si substrates were labeled as S_1_, S_2_, and S_3_, corresponding to the processing/controlled sublimation temperatures of 600, 700, and 800 °C, respectively.

### 2.4. Characterization Techniques

The distinct film thicknesses were measured using a profilometer (Dektak, Bruker, MA, USA). Their structural characteristics were analyzed using X-ray diffraction (XRD; INEL EQUINOX 2000) with Cu-Kα radiation (wavelength, λ = 0.15406 nm). The chemical composition and sample features were evaluated using X-ray photoelectron spectroscopy (XPS; K-Alpha model, Thermo Fisher Scientific, Waltham, MA, USA) with an Al–Kα monochrome X-ray source (for calibration, the C1s peak was used as the reference). The film’s optical properties were characterized via photoluminescence (PL) measurements (Hamamatsu photomultiplier attached to a SPEX spectrometer with a focal length of 50 cm); the film’s PL spectra were recorded with the 325 nm emission line of a 200 mW He–Cd laser. The surface morphologies of the produced films were examined using field-emission scanning electron microscopy (FESEM; JSM-7800F, JEOL, Tokyo, Japan) with an acceleration voltage of 3–5 kV. All of the characterizations were carried out at room temperature.

## 3. Results

### 3.1. X-ray Diffraction

**Figure 1:**
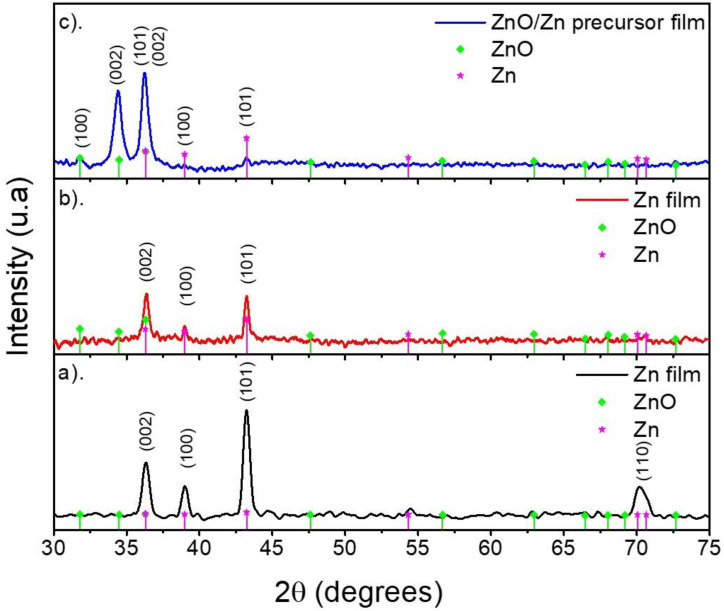
Comparison between the XRD patterns of the precursor film on quartz in the (**a**) early, (**b**) middle, and (**c**) final processing stages.

Figure 1 shows the comparison between the diffraction pattern corresponding to ZnO/Zn precursor films on the quartz substrate over the time of processing. In the early stage of precursor layer processing (Figure 1a), peaks located at 36.48, 39.14, 43.31, and 70.77 degrees were identified, which were in good agreement with the diffraction exhibited by the (002), (010), (011), and (013) planes for the hexagonal zinc phase, respectively (reference code: COD-00-04-0831) [36]. We could observe the absence of ZnO peaks, which implied that the as-deposited Zn layer was obtained without additional phases. Then, in the middle stage of precursor layer processing (Figure 1b), an increment in the relationship of (002) plane intensity and that exhibited by the other planes was observed. This could be attributed to the presence of ZnO due to the diffraction of the plane (101) located at 36.49 degrees. The previous suggestion was reinforced by the final stage of precursor layer processing (Figure 1c) because the diffraction of the planes (100), (002), and (101) attributed to hexagonal wurtzite ZnO could be distinguished based on their locations at 31,82, 34.33, and 36.49 degrees, respectively (reference pattern: COD-00-36-1451). The diffraction of the (002), (010), and (011) planes (previously attributed to Zn) suggested the co-existence of metallic zinc and ZnO. The presence of both phases confirmed the existence of the Zn/ZnO precursor layer (see Figure S1).

**Figure 2:**
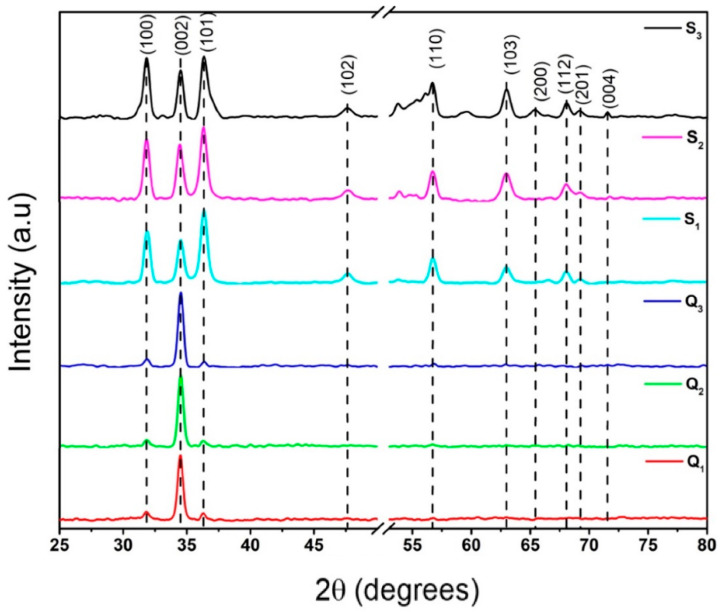
XRD patterns of the ZnO films—supported on quartz (Q_1_–Q_3_) and Si (S_1_–S_3_) substrates—obtained through the sublimation of ZnO from the ZnO/Zn precursor films at different temperatures.

Figure 2 shows the XRD patterns of all samples prepared on both the Si and the quartz substrates. These results suggest that the polycrystalline wurtzite ZnO films were formed without metallic Zn peaks in the XRD patterns of the high-temperature-oxidized ZnO/Zn precursor films. This indicates that the raw Zn films were completely converted into ZnO by the applied process. The XRD patterns of the samples that were grown on both the quartz and the Si substrates show three main characteristic peaks corresponding to the diffraction of the (100), (002), and (101) planes of wurtzite ZnO [36]. Additionally, for the samples with the precursor layer deposited on the Si substrate, the peaks located at 47.54, 57.25, 63.15, 66.75, 67.85, 68.97, and 72.66 degrees were found, and these were correlated with the diffraction of (102), (110), (103), (200), (112), (201), and (004), respectively. This observation corroborates the presence of the wurtzite ZnO phase and suggests that the substrates have a strong influence on the growing process. The preferential growth of planes could be related to a modification in morphology in the ZnO structures [37]. For the films deposited on quartz substrates (Q_1_, Q_2_, and Q_3_), the peak corresponding to the (002) plane showed a higher intensity than those of the other two visible peaks, indicating the preferential orientation of ZnO crystallites along the ZnO c-axis perpendicular to the substrate plane [38]. This preferential orientation can be attributed to the self-texturing phenomenon [39]. In contrast, the samples grown on the Si substrates did not show any preferential orientations. In this case, nanocrystalline ZnO films were obtained because of the influence of the crystalline Si substrate on the films.

The structural characteristics of the films (Q_1_–S_3_) were extracted from the peak corresponding to the (002) plane. The crystallite size was calculated using the Scherrer equation [40], expressed as:

(1) $$D=\frac{k\lambda }{\beta \cos\theta }$$

where λ is the wavelength of the analyzed peak (λ = 1.54059 Å); k is a constant with a value of 0.94; β is the full width at half maximum (FWHM) of the peak; θ is the diffraction angle of the peak. The corresponding results are presented in Table 1, which shows that the average size of the sample crystallites grown on the Si substrates was slightly smaller (~14 nm) than those grown on quartz substrates (~16.8 nm). For the samples on quartz, the size of the crystallite decreased with the sublimation temperature, while for the samples on silicon substrates, the behavior was the opposite. Thus, it increased with respect to the sublimation temperature (see Table 1). The position of the (002) peak was approximately the same for both of the substrates, implying that the films were in a relaxed condition.

### 3.2. X-ray Photoelectron Spectroscopy

**Figure 3:**
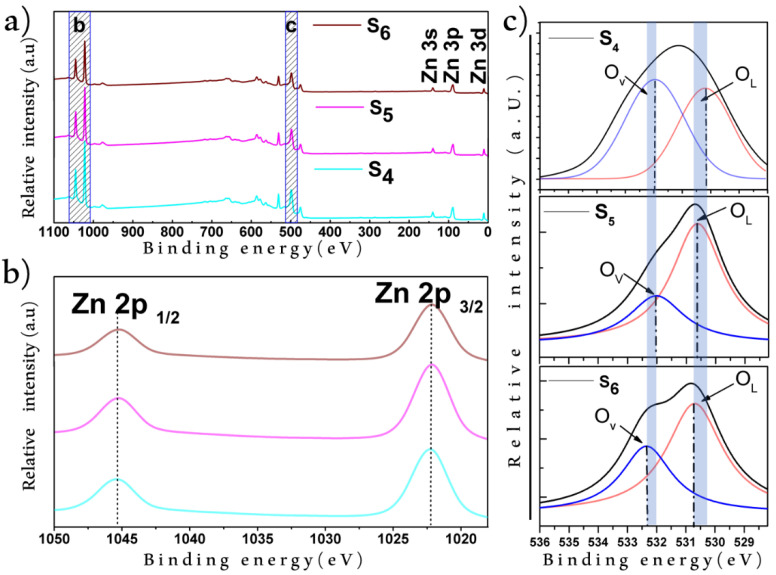
Comparison between the XPS spectra of the ZnO nanofilms supported on the Si substrate after controlled sublimation at 600 (S_1_), 700 (S_2_), and 800 °C (S_3_): (**a**) complete spectra; (**b**) spectra in the 1050–1020 eV binding energy range; (**c**) spectra in the 536–528 eV binding energy range.

According to the XPS measurements, the stoichiometry of the samples was in good agreement with the phase identified in the XRD results. In addition, the samples were obtained with a high control in purity due to O and Zn being the unique elements present in the ZnO film (see Figure 3a). The comparison between the XPS spectra of the Zn on the Si substrate after thermal oxidation processed at 600, 700, and 800 °C revealed no significant shifting in the Zn2p_3/2_ and Zn2p_1/2_ energy bands (EBs), which were placed at 1022.8 and 1045.8 eV, respectively (Figure 3b). The constant difference of 23 eV between the Zn2p_3/2_ and Zn2p_1/2_ bands suggested a dense population in Zn^2+^ [41]. Notice that, typically, Zn presents a smaller energy value in the Zn2p_3/2_ EB than that observed in our samples; this confirms that samples have oxygen-deficient ZnO_1-x_ and that Zn is present only in an oxidized state [42]. Thus, the presented method allowed us to obtain a full Zn-oxidized film even when we used a Zn layer as a precursor in the synthesis. In addition, it is known that the O1s EB is composed of oxygen vacancies (O_V_), chemisorbed oxygen (O_C_), and lattice oxygen vacancies (O_L_) [43,44]. Thus, the components of the O1s EB were determined by applying a Gaussian fitting on the XPS spectra (Figure 3c); for the S4 sample, the O_L_ and O_V_ were found at 530.17 and 531.97 eV, respectively. For the S5 sample, the O_L_ was located at 530.66 eV and the O_V_ was located at 532.02 eV, while in sample S6, the same O1s EB components (O_L_ and O_V_) were found at 530.67 and 532.37 eV, respectively. Note that in all samples, both O_L_ and O_V_ were found in different intensities; this could be related to the synthesis temperature, since it was observed that temperature had a direct influence on the structural properties of ZnO [45]. The variation in O_V_ intensity demonstrated different oxygen vacancy concentrations in the samples. To measure this concentration, the percentage contributions of oxygen vacancies in the O1s EB were calculated as 59.82%, 38.69%, and 41.92% for S4, S5, and S6, respectively. This suggested that the treatment at 600 °C promoted the highest concentration of oxygen vacancies on ZnO. As can be seen in Figure 3c, the O1S shifted across the samples, and it was proposed that the change in oxygen-deficient states was responsible for this shifting [46].

### 3.3. Photoluminescence

**Figure 4:**
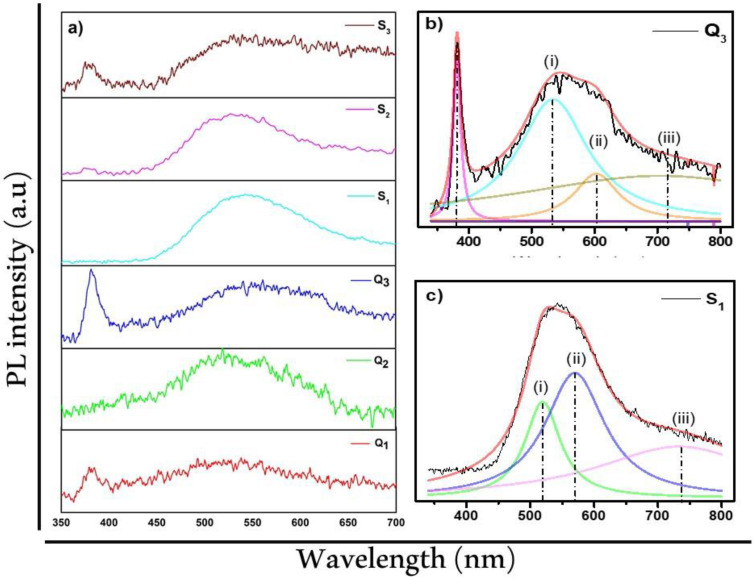
PL spectra of (**a**) the nanoporous ZnO films synthesized at different temperatures on quartz and Si substrates, (**b**) the ZnO film on the quartz substrate when annealed at 800 °C (Q_3_), and (**c**) the ZnO film on the Si substrate when annealed at 600 °C (S_1_).

Typically, photoluminescence measurements are used to obtain the defects present in a semiconductor by studying its optical properties. The PL spectra obtained at room temperature for ZnO were integrated with two peaks: (1) the UV emission (originated from excitonic recombination corresponding to the near band-gap emission of ZnO) and (2) the emission at the deep level (DL), which originated from the presence of several intrinsic defects, which are also called native defects [47]. These defects can be referred to as zinc interstitial (Zn_i_), zinc vacancy (VZ_n_), oxygen interstitial (O_i_), oxygen vacancy (O_V_), oxygen antisite (O_Zn_), and Zn antisite (ZnO) defects [48], which originate from different emissions in the visible range [12]. ZnO is unlikely to be stable under equilibrium conditions due to the high formation energies, even in a Zn-rich atmosphere. Zni and O_V_ give rise to free (i.e., donor) electrons in ZnO crystals, while V_Zn_, O_i_, and O_Zn_ consume free (i.e., acceptor) electrons. The relative content of donors and acceptors determines the semiconductor property of ZnO [2]. Although many investigations have been carried out to explain the luminescent properties of ZnO, a clear reason for how the presence of defects is related to the emissions in the visible range still needs to be established. One of the emissions that has been studied the most by different investigations and whose explanation is still uncertain is the emission in green (peak i in Figure 4). Researchers have tried to explain it in powders, films, nanostructures [48,49,50,51,52], and single crystals [53]. In addition, the relationships of this emission have been linked with the effect of the atmosphere and annealing temperature [54], the presence of porosity on the surface [11], increase in deposition power through RF sputtering [12], and calculations of the native defect levels in ZnO [55].

Different theories have been established to try to explain the green emission. One of them (the one that has been most accepted) said that the emission is generated from the presence of oxygen vacancies [56,57]. The green emission has also been attributed to oxygen vacancies and interstitial zinc [58]. In some other hypotheses, importance is given to transitions related to intrinsic defects, such as donor–acceptor transitions [59], as well as the presence of impurities, such as the influence of dopant on ZnO [13,14,15,60]. Figure 4 presents the PL spectra of the porous ZnO films deposited on quartz and silicon, as well as the Gaussian deconvolutions of the PL spectra of sample S3 (Figure 4b) and sample S4 (Figure 4c). We will start by describing the effects of the temperature and substrate on the UV emissions from excitonic recombination corresponding to the near band-gap emission of ZnO. As we can see in Figure 4a, at low annealing temperatures, there was no presence of the said band (S2, S4, and S5). However, when the samples were annealed at high temperatures, there was the presence of the said band in the case of sample S3. This is in good agreement with the DL band; Q_3_ and S_3_ confirmed the crystalline quality of the film under the conditions that were obtained, as shown by the results of XRD. Note that (iii) in Figure 4b,c is a noise related signal. We could see that the emission at the deep level (DL) was present with great intensity in the samples that are annealed at the temperatures of 600 and 700 °C, which indicated that the density of defects was higher. In these samples, when the deconvolution of samples S3 and S4 was carried out, it could be seen that the emission corresponding to oxygen vacancy (V_O_) was present in both samples with greater intensity in S4 compared to S3; likewise, an emission in yellow could be observed (peak ii in Figure 4). We can comment that the density of Vo was strongly influenced by the morphology present on the surface of the films. Sample S3 exhibited a multiple-stacked porous structure, but in the case of sample S4, the greatest contribution to the emission could be related to the formation of hexagonal nut-like nanostructures. Therefore, the multiple-stacked porous structure played an important role in the emission in green (peak i in Figure 4). However, when there were hexagonal nut-like nanostructures, a high emission of the deep level was present in addition to the high concentration of oxygen vacancies (according to the XPS results shown in Figure 3c).

### 3.4. Field-Emission Scanning Electron Microscopy

**Figure 5:**
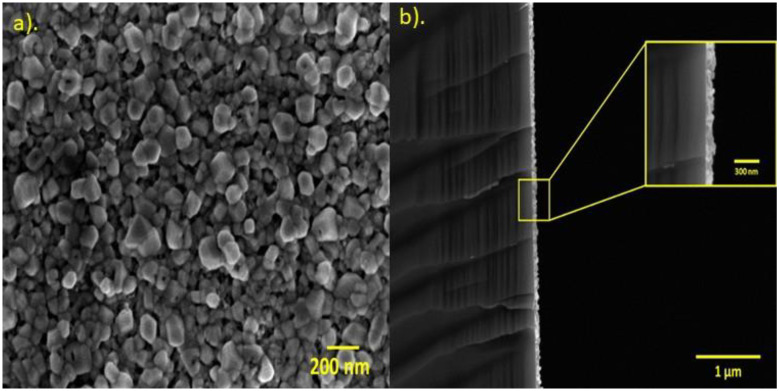
(**a**) The SEM images of the surface morphology of the Zn film; (**b**) the cross-sectional SEM image of the Zn film on the silicon substrate.

The as-deposited Zn film was analyzed with SEM to determine the morphology on the surface. Figure 5a shows a frontal view of the sample, where the Zn film was composed of irregular features with a low-porosity degree. To corroborate the possible existence or absence of pores in the Zn film, a cross-sectional image was recorded through the detection of backscattered electrons in the SEM (Figure 5b). The known difference in contrast (due to atomic mass variation between the elements in the sample) could allow one to distinguish the substrate (darkest area) from the Zn (brightest area). In addition, the pores should be depicted in the SEM images of Zn as intense dark zones. No zones with this characteristic were found (see insert in Figure 5b). Thus, the results suggest that the Zn film was obtained as a highly homogeneous film without porosity. The previous idea was demonstrated through a closer examination (see Figure S2).

Based on cross-section analysis, the thickness of films was determined finding an average value of 90 ± 8.6 nm for the Zn films. On the other hand, by analyzing the cross-section of ZnO nanoporous films (obtained after treatment described in Section 2.3) the measured thickness was 165 nm ± 11.6 nm. These values were corroborated by profilometry for the case of samples deposited on quartz, and for the samples on silicon substrates, by ellipsometry. The thicknesses obtained for all the samples are summarized in Table 2. The increment in the thickness size from Zn to ZnO films could be attributed to oxygen incorporation.

**Figure 6:**
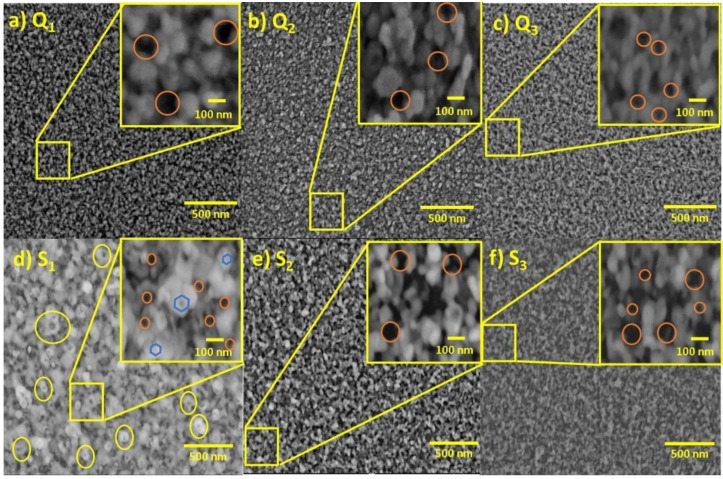
Comparison among the surfaces of the samples imaged with SEM after the precursor layer was processed at (**a**) 600, (**b**) 700, and (**c**) 800 °C on quartz substrates and processed at (**d**) 600, (**e**) 700, and (**f**) 800 °C on Si substrates. The inserts show magnified sections of the samples.

Figure 6 shows a frontal-view image obtained through SEM of the as-processed samples after the sublimation procedure. The images of the nanoporous ZnO film surfaces deposited on the quartz substrates (Figure 6a–c) and Si substrates (Figure 6d–f) indicated that the film surfaces were homogeneous and indicated the presence of porosity. Thus, a clear change in porosity was observed after the treatment on the Zn film (see Figure S1). In addition, particles with a size between 80 and 100 nm were observed. These features are typically expected on surfaces grown by DC sputtering [61]. Furthermore, the surfaces of the ZnO/Zn precursor film with controlled sublimation temperatures (at 600, 700, and 800 °C) were supported on quartz substrates (Figure 6a–c, respectively). Despite the great similarity observed between the samples, differences in the density of gaps (spaces between the previously mentioned particles) and their average size can be seen in Figure 6 (indicated by orange circles). Thus, it can be said that the pore distribution obtained by the quartz substrate was different from that of the samples supported on Si substrates (Figure 6d–f, for 600, 700, and 800 °C respectively). The previous observation suggests that the substrates influenced the film morphologies. The reader is referred to Figure S3 in the supporting information to closely see the differences between the porosities obtained in the samples (see the details of the methodology for preparation and sample imaging in Section S1).

Notably, the ZnO films supported on quartz substrates showed porous surface structures with high densities. After the low-temperature processing at 600 °C, relatively large and nearly spherical particles were observed on the film surfaces, whereas after the high-temperature processing at 800 °C, fiber-like structures were observed on the film surfaces on both of the substrates (Figure 6). In particular, the surface of sample S_1_ showed a uniform distribution of hexagonal nut-like nanostructures with a cavity at the center, indicated by yellow circles in Figure 6d. Such structures are clearly distinguishable because of their large sizes, and their formation can be attributed to the orientation of the substrate [23] and the processing temperature of the film [62]. As the Si wafers belong to the cubic crystalline system and the ZnO belongs to the hexagonal system, the lattice mismatch between ZnO and Si could be highly responsible for the hexagonal structures [23]. For samples S_2_ and S_3_, hollow-like particles were observed, but only at higher magnifications (see Figure S3). The different ZnO film microstructures that were observed in this study resulted from the amorphous and crystalline substrates.

In the next section, a qualitative mechanism based on these results is proposed to explain the formation of porous ZnO films. The reason that hollow structures become smaller as temperature increases could be attributed to the increment in the partial pressure of Zn [31]. Even though the sublimation method is not a novel proposal, the use of the Zn/ZnO precursor layer to synthesize a uniform porous surface with this method constitutes the novelty of the present work. In addition, the comparison of two different substrates (Si and quartz) opens the door to discussion about the explanation for the morphological differences in the nanoporous ZnO films promoted by each kind of substrate. Exploring the influence of the substrate on the morphology of nanostructured materials is important because it is linked with surface properties, which have a direct relationship with surface-based applications [63]. As the methodology for synthesizing nanoporous ZnO films is still the sublimation method, all of the advantages, such as high homogeneity, accessibility, ease of implementation, and high reproducibility [33], remain in the present proposal.

### 3.5. Mechanism of Nanoporous ZnO Film Formation

**Figure 7:**
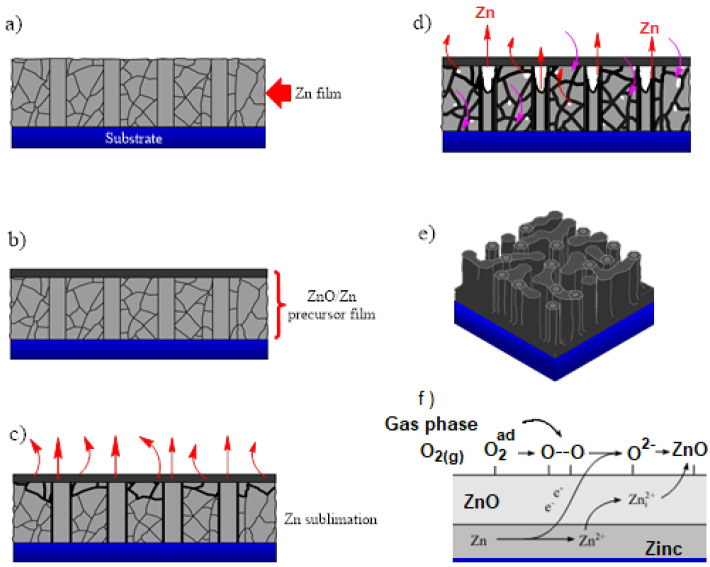
Scheme of the proposed mechanism during the formation of nanoporous ZnO films through the sublimation of Zn from the ZnO/Zn precursor film.

The mechanism for the nanoporous ZnO film formation is driven by the high partial pressure of Zn generated by the processing temperature and stabilized by the ZnO/Zn precursor films; the stabilization of the partial pressure of Zn can be advantageously used to produce high-quality epitaxial ZnO films through molecular beam epitaxy. According to Anthrop and Searcy, zinc vaporization can be controlled in Knudsen cells at high temperatures (1051–1068 °C) by the presence of minute quantities of water vapor or CO_2_ [31]. The partial pressure stabilization of zinc can also be reached when a mixture of ZnO, SnO_2_, and graphite powders is heated at 1150 °C under Ar gas, as was demonstrated by Gao et al. [32], who studied a procedure for producing hollow ZnO shells of controlled dimensions in the nanosize range. These authors produced hexagonal drums and spherical/hemispherical shells when the reaction products were collected downstream on alumina substrates at 300–500 °C. Recently, Zacarias et al. explained those results based on the vaporization/sublimation of Zn core in the ZnO cage/shell structure [64]. These studies demonstrate that the ZnO/Zn structure stabilized the sublimation of zinc. In this work, the production of nanoporous ZnO films can be explained by the role played by the ZnO/Zn precursor film in controlling the Zn sublimation. To produce the precursor film, a ZnO film of a few nanometers in thickness was grown on the Zn film through thermal oxidation at low temperatures. Figure 7 illustrates the details of the mechanism involved during the formation of nanoporous ZnO films. In Figure 7a, a scheme of Zn film after the sputtering process is depicted. In this step, irregular particles of different sizes are obtained in the sample. After a thermal oxidation process under chromatographic N_2_, a thin layer of ZnO appears on the upper side of the Zn film (see Figure 7b).

The Zn atoms from the zinc film migrate towards surface reaction sites located on the ZnO crystallite seeds. The sublimation rate can also be enhanced by increasing the processing temperature, the microstructure of the ZnO nanofilm, and the concentration of various oxidizing gas species—H_2_O or CO_2_. The formation of nanopores is the result of the sublimation of Zn through the film’s structural defects or the grain boundaries located in the ZnO/Zn precursor film; the zinc migration can be enriched by increasing the processing temperature due to the high surface diffusivity of Zn over the ZnO seeds (see Figure 7d). The ZnO crystallite formation induces ZnO surface fracture due to the polycrystalline nature of the ZnO/Zn precursor film. The pore diameter is related to the initial thickness of the Zn film, the processing temperature, and the substrate’s nature. The presence of hexagonal features on the film surface reveals the incidence of the wurtzite phase of the ZnO films (see Figure 7e). In Figure 7f, a scheme is proposed to illustrate the possible path followed by Zn to obtain ZnO in the present procedure. Thus, the Zn diffuses through the ZnO/Zn precursor film to interact with oxygen atoms (from the atmosphere and into the process) to leave the formation of ZnO structures. In addition, the fact that the melting point of Zn is under 450 °C seems to be an important property for obtaining ZnO micro-/nano-structures in thermal-oxidation-related processes [36]. The importance of this property in the above-described mechanism is under discussion. Even though similar works have been reported [23,65], this work contributes novelty by giving experimental evidence of the importance of a ZnO/Zn precursor film for the obtention of a porous ZnO layer from pure Zn deposition, which could be a strategy for increasing the accessibility of the sublimation methodology for nanoporous ZnO film fabrication.

## 4. Conclusions

In this study, a reliable methodology was developed for obtaining nanoporous ZnO films via Zn sublimation in a ZnO/Zn bilayer. Using the proposed method, nanoporous ZnO films with hexagonal nut-like particles that had a diameter of approximately 70 nm and high surface-to-volume ratios were produced. Such films are highly desirable for advanced gas-sensing applications. The XPS and PL measurements showed that prevalent structural lattice defects were produced by Vo, and they were generated during the controlled sublimation process. On the surfaces of the samples supported on amorphous quartz substrates, porous particles of different geometries were formed. In contrast, the films supported on Si substrates exhibited well-defined hexagonal nano-shapes at a sublimation temperature of 600 °C; this indicates that the Si substrate structure directly influenced the surface porosity of the ZnO thin films produced via Zn sublimation at higher processing temperatures.

## Figures and Tables

**Table 1:** Summary of the structural characteristics of ZnO obtained from the XRD patterns—(002) plane—of the ZnO films formed on quartz and Si substrates and processed at different temperatures.

Sample	Sublimation Temperature (°C)	2θ (°)	FWHM	D (nm)
Q_1_	600	34.48	0.47 ± 0.03	17.55 ± 0.02
Q_2_	700	34.52	0.49 ± 0.06	16.82 ± 0.04
Q_3_	800	34.52	0.49 ± 0.02	16.88 ± 0.03
S_1_	600	34.52	0.61 ± 0.04	13.56 ± 0.05
S_2_	700	34.48	0.59 ± 0.03	14.16 ± 0.07
S_3_	800	34.50	0.51 ± 0.06	16.18 ± 0.02

**Table 2:** Comparison between the experimentally measured thicknesses of the porous ZnO films.

Sample	Thickness (nm)
Q_1_	159.65
Q_2_	163.4
Q_3_	153.1
S_1_	162.2
S_2_	166.89
S_3_	164.64

## Data Availability

The data presented in this study are available on request from the corresponding author. The data are not publicly available due to privacy concerns.

## Supplementary Materials

The following supporting information can be downloaded at: https://www.mdpi.com/article/10.3390/ma15165509/s1, Figure S1: The side view of Zn/ZnO precursor film imaged by STEM (in bright-field mode) after the complete formation process; Figure S2: As-deposited Zn film before the oxidation process and imaged by STEM (in bright-field mode); Figure S3: Samples were processed at (**Q1**) 600 °C, (**Q2**) 700 °C, and (**Q3**) 800 °C on quartz substrates and imaged by STEM (FIB-processed). Samples were processed at (**S1**) 600 °C, (**S2**) 700 °C, and (**S3**) 800 °C on Si substrates imaged by STEM (FIB-processed). The yellow circles point to the porous of the ZnO film.
