# Supplementary material for: Synthesis and Characterization of Nanoporous ZnO Films by Controlling the Zn Sublimation by Using ZnO/Zn Precursor Films
Source: Materials (Basel). 2022 Aug 11;15(16):5509. doi: 10.3390/ma15165509 (PMC9412365; doi:10.3390/ma15165509)
Supplement: Supplementary file 1 [file materials-15-05509-s001.zip › materials-1657349-supplementary.pdf]

Supporting Information

Synthesis and characterization of nanoporous ZnO films by controlling the Zn sublimation by using ZnO/Zn precursor films

Y.M. Hernández-Rodríguez[1]*, P. Lopez-Salazar[2], G. Juarez-Diaz[3], G. Romero Paredes[4], and R. Peña-Sierra[4]

[1]Programa de Doctorado en Nanociencias y Nanotecnología, CINVESTAV-Instituto Politécnico Nacional, Av. Instituto Politécnico Nacional 2508, Ciudad de México, C.P. 07360, México.
[2]Centro de Investigación en Dispositivos Semiconductores, Instituto de Ciencias, Benemérita Universidad Autónoma de Puebla, Ciudad Universitaria, Puebla, Pue. 72570, México.
[3]Facultad de Ciencias de la Computación, BUAP, Puebla, Pue. 72570, México.
[4]Departamento de Ingeniería Eléctrica, Sección de Electrónica del Estado Sólido (SEES) CINVESTAV-IPN, Av. Instituto Politécnico Nacional 2508, Ciudad de México, C.P. 07360, México.

*Correspondence: yazmin.hernandez@cinvestav.mx

S1. FIB preparation for imaging the samples by transmission electron microscopy (TEM).

The ZnO films were prepared by the focus ion beam (FIB) technique in a JIB-4500 MultiBeam equipment with a gallium (Ga) liquid metal ion source to study the nanoporous structures in the samples with higher detail. Then, both samples (those grown on Si and those grown on quartz) were imaged by scanning transmission electron microscope (STEM) in low and high (HR-STEM) resolution modes in a JEM-ARM200F microscope.

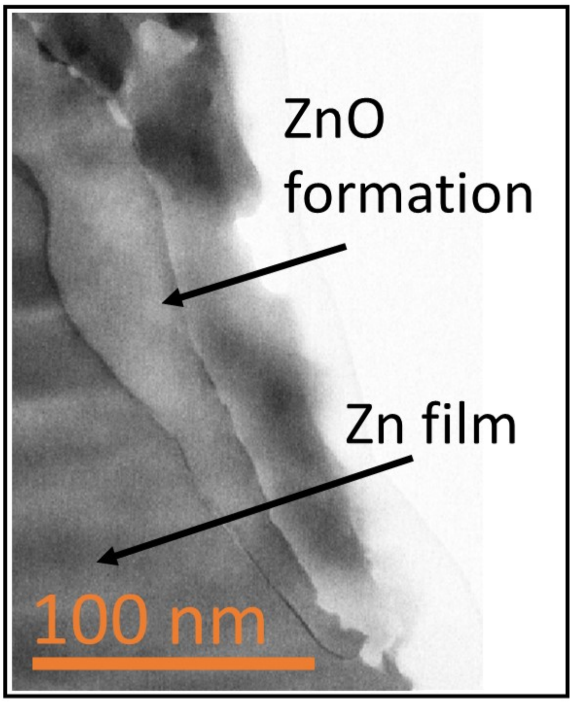

Figure S1. The side view of Zn/ZnO precursor film imaged by STEM (in bright-field mode) after the complete formation process.

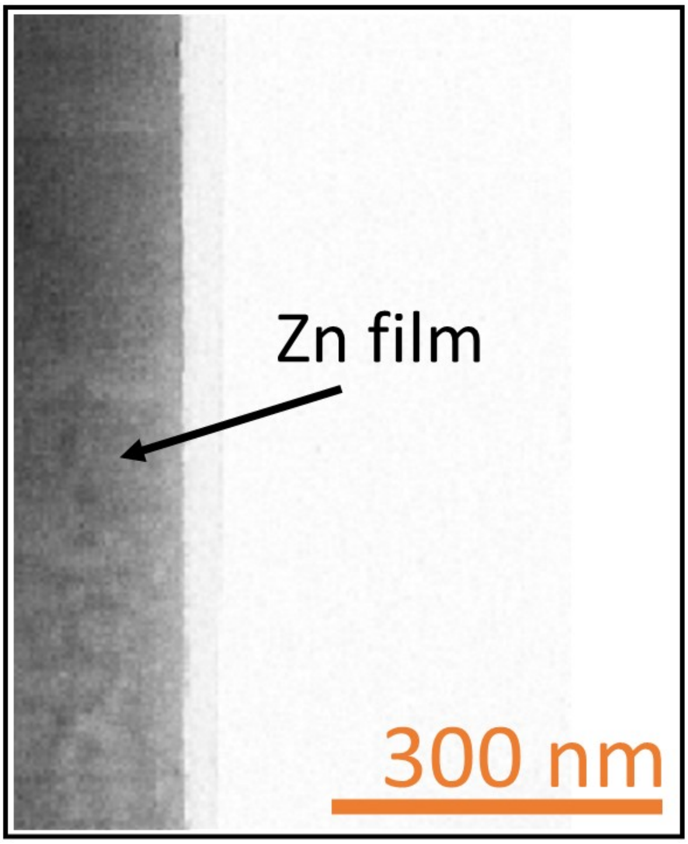

Figure S2. As-deposited Zn film before the oxidation process and imaged by STEM (in bright-field mode).

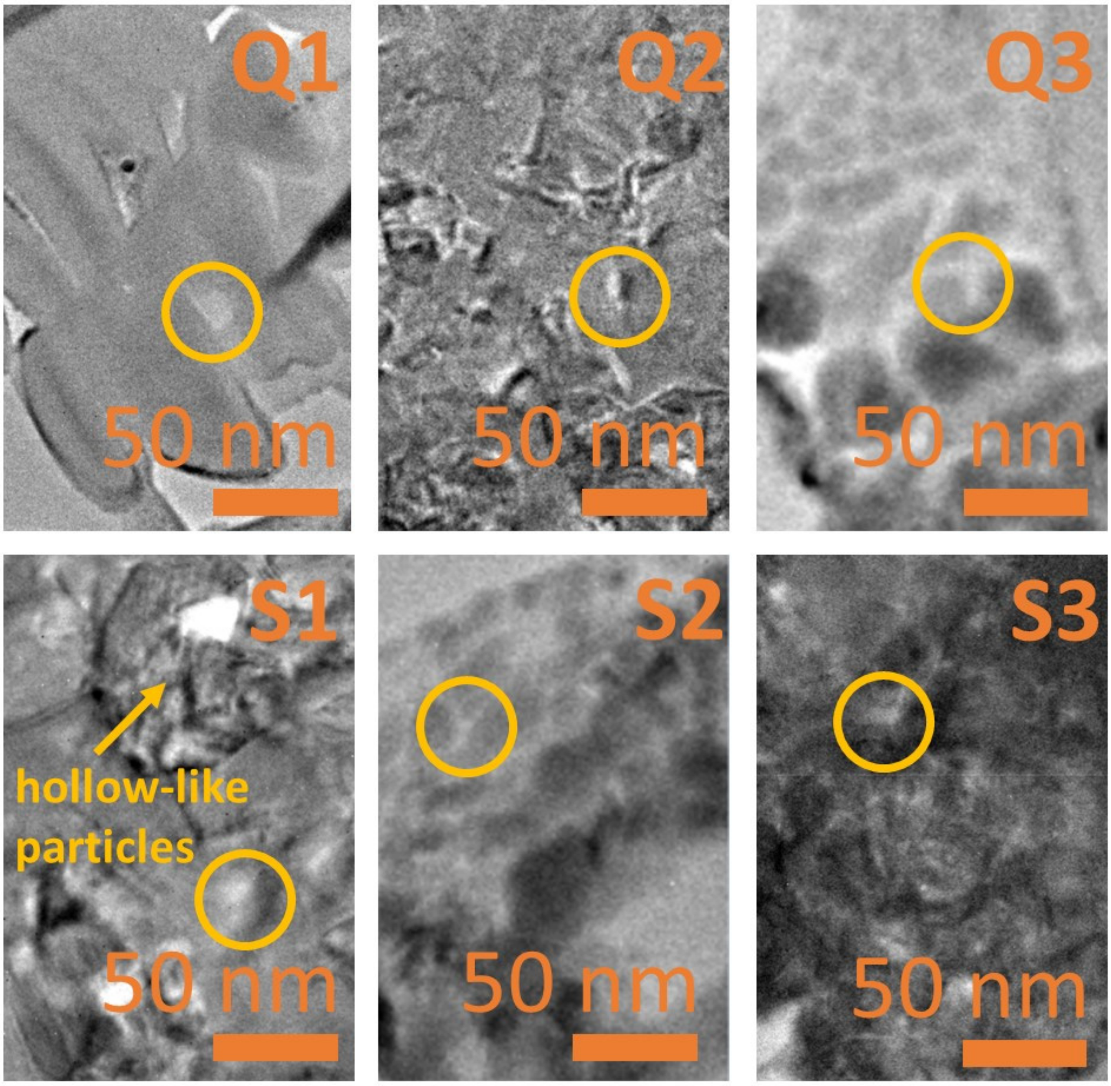

Figure S3. Samples were processed at (**Q1**) 600 °C, (**Q2**) 700 °C, and (**Q3**) 800 °C on quartz substrates and imaged by STEM (FIB-processed). Samples were processed at (**S1**) 600 °C, (**S2**) 700 °C, and (**S3**) 800 °C on Si substrates imaged by STEM (FIB-processed). The yellow circles point to the porous of the ZnO film.
